# Preliminary Outcomes of Zone 2 Thoracic Endovascular Aortic Repair Using Castor Single-Branched Stent Grafts: A Single-Center Experience

**DOI:** 10.3390/jcm12247593

**Published:** 2023-12-09

**Authors:** Antonio Rizza, Giancarlo Trimarchi, Silvia Di Sibio, Luca Bastiani, Michele Murzi, Cataldo Palmieri, Ilenia Foffa, Sergio Berti

**Affiliations:** 1 Fondazione Toscana Gabriele Monasterio, 54100 Massa, Italy; disibio@ftgm.it (S.D.S.); luca.bastiani@cnr.it (L.B.); michele.murzi@ftgm.it (M.M.); palmieri@ftgm.it (C.P.); ilenia.foffa@cnr.it (I.F.); berti@ftgm.it (S.B.); 2 Department of Clinical and Experimental Medicine, University Hospital of Messina, 98121 Messina, Italy; giancarlo.trimarchi18@gmail.com; 3 Institute of Clinical Physiology, National Research Council, 54100 Massa, Italy

**Keywords:** aortic arch pathologies, type B aortic dissections, endovascular approach, single-branched stent graft, safety

## Abstract

In the context of thoracic endovascular aortic repair (TEVAR), the reconstruction of the left subclavian artery (LSA) has emerged as a crucial component in establishing a sufficient proximal landing zone. However, the technical difficulty of these procedures raises the possibility of endoleaks and neurological consequences. Single-branched stent grafts offer good anchoring and LSA flow for these patients. This study evaluates the feasibility of utilizing novel single-branched stent grafts in the treatment of distal aortic arch disease, identifying good results in the short and medium term. From September 2019 to March 2023, TEVAR and revascularized LSA were performed on ten patients at the Ospedale del Cuore—FTGM in Massa, Italy, using Castor single-branched thoracic aortic stent grafts (Microport Medical, Shanghai, China). The authors’ first findings demonstrated that, after an average follow-up of one year, the Castor branching aortic stent graft system was safe and achieving an appropriate proximal landing zone and maintaining sufficient LSA perfusion was possible. With regard to the endovascular treatment of distal aortic arch diseases, this product offers a compelling substitute for surgery. For the purpose of assessing the long-term effectiveness of this approach, the follow-up period should be extended.

## 1. Introduction

Aortic arch diseases include dissection, aneurysm, intramural hematoma, and penetrating aortic ulcer (PAU). Traditionally, aortic arch pathologies have required surgical repair to redirect the blood flow to the true lumen and eliminate the false lumen, prevent organ malperfusion, and minimize brain damage. However, despite recent advancements in surgical therapeutic techniques, surgical mortality and morbidity remain high, particularly among elderly patients with significant comorbidities [1,2]. Over the past few years, thoracic endovascular aortic repair (TEVAR) has become increasingly popular. Since the endovascular method is less intrusive than open surgery and does not require aortic cross-clamping or circulatory support, it has better outcomes in terms of perioperative mortality, neurological injury, and length of hospital stay [3,4]. Accordingly, endovascular therapy could be a useful therapeutic choice for reducing surgical trauma in patients who are at high risk; TEVAR was created to treat several types of aortic lesions and has been used more often for patients with type B aortic dissections (TBADs) since reports of its positive short-term outcomes in 1999. It is currently being investigated as an endovascular therapy for lesions in the arch and even in the ascending aorta [5,6,7]. The aorta is currently divided based on the landing zone of the proximal and distal attachments using the Ishimaru classification, with a focus on the endovascular treatment of aortic arch lesions [8]. The left subclavian artery (LSA) must often be covered in order to create a sufficient proximal landing zone. The blood flow can be restored to the LSA using a variety of methods, such as carotid-subclavian bypass, fenestration, and parallel (chimney) stents. However, these procedures are technically challenging, which raises the possibility of endoleaks and neurological issues. Stent grafts with a single branching offer these patients adequate anchoring and LSA flow. In this study, the authors documented their initial expertise in treating distal aortic arch illness using a Castor single-branched stent graft, focusing on its efficacy, safety, and short- and medium-term outcomes.

## 2. Materials and Methods

### 2.1. Patient Population

**Figure 1:**
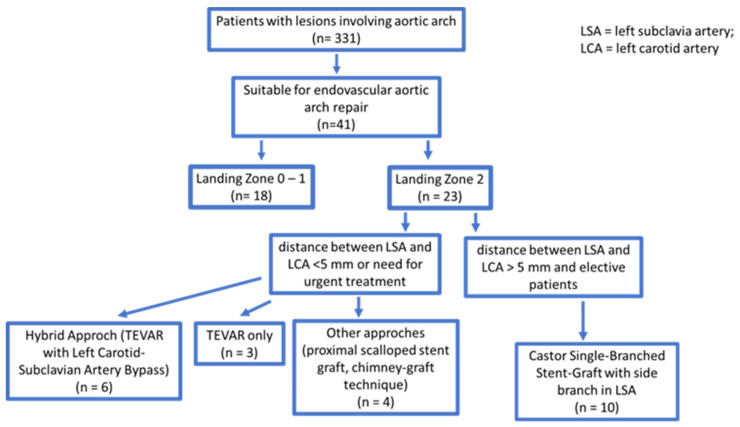
Flow chart of the study.

From September 2019 to March 2023, TEVAR with LSA revascularization was performed on 10 consecutive patients using Castor single-branched thoracic aortic stent grafts (Microport Medical, Shanghai, China) at the Ospedale del Cuore—FTGM, Massa, Italy. The screening process is depicted in Figure 1.

The study was conducted according to the guidelines of the Declaration of Helsinki and was approved by the Ethics Committee of the hospital (protocol code 21728; date of approval: 4 November 2022).

The inclusion criteria were the following:

1. Adults older than 18 years of age, regardless of gender, excluding pregnant women;
2. Individuals diagnosed with distal aortic arch diseases, such as aortic dissection (AD), penetrating aortic ulcer (PAU), thoracic aortic aneurysm (TAA) exceeding 5.5 cm in diameter, or cases in which the aorta demonstrated rapid growth (greater than 1 cm per year);
3. The distance between the proximal end of the aortic lesion and the ostium of the LSA had to be less than 15 mm;
4. Assurance that the aortic disease did not affect the left common carotid artery (LCCA), with a minimum distance from the proximal aortic lesion to the LCCA ostium of more than 15 mm;
5. The distance between the origin of the left vertebral artery (LVA) and the foramen of the LSA had to be greater than 25 mm;
6. A minimum separation of 5 mm between the ostium of the LCCA and the ostium of the LSA was necessary; and
7. A maximum aortic arch diameter of 40 mm.

The exclusion criteria were the following:

1. Patients with connective tissue diseases, such as Marfan syndrome and Ehlers–Danlos syndrome;
2. Individuals with severe organ diseases which would hinder surgery or anesthesia;
3. Cases of Stanford type A aortic dissection;
4. Patients with an aberrant right subclavian artery;
5. Those in whom the diameter of the external iliac artery or common femoral artery (FA) was less than 7 mm; and
6. Individuals with allergies to nitinol or iodine contrast agents.

Prior to the surgical procedure, all patients underwent high-resolution computed tomography angiography (CTA) of the aorta.

### 2.2. Stent Graft

**Figure 2:**
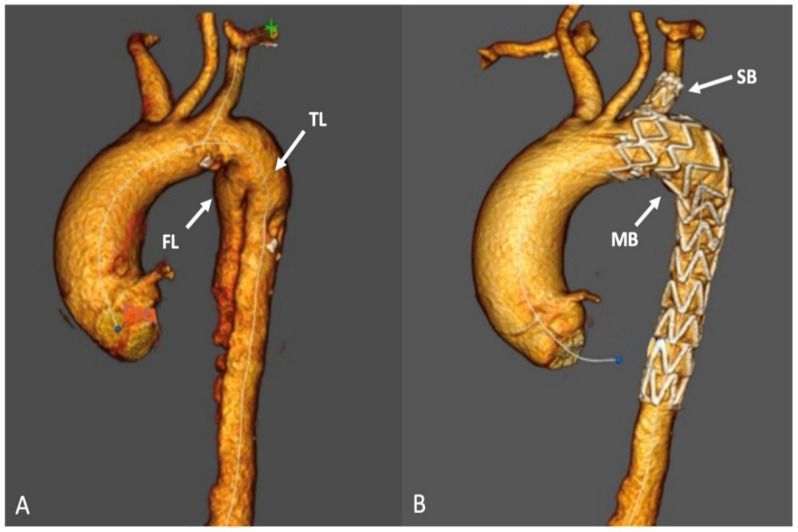
(**A**) Type B aortic dissections with false lumen (FL) and true lumen (TL) and (**B**) Castor single-branched stent grafts for type B aortic dissections. The stent consists of a main body (MB) and a solitary branch (SB).

Every patient received treatment using a Castor single-branched stent graft manufactured by MicroPort Endovascular in Shanghai, China. The stent grafts were constructed from nitinol and polyester materials, and consisted of a main body (MB) and a solitary branch (SB) (Figure 2). The deployment of the device was accomplished with a 24-F delivery sheath. Four parameters were chosen according to preoperative CTA measurements: the proximal and distal end diameters of the MB, the diameter of the distal end of the SB, and the distance from the ostium of the solitary branch to the proximal end of the main body.

### 2.3. Stent Graft Deployment

All surgeries were performed under general or local anesthesia. For the procedure, the left and right femoral arteries were punctured and two 6-French (FR) introducers were placed in each vessel. Two ProGlide preclosure suture devices (Abbott Vascular Devices, Santa Clara, CA, USA) were placed at the right arterial puncture site at a 45° angle to each other for optimal access site closure after the implant. The right 6-Fr introducer was then exchanged for a 10-Fr introducer using a standard 0.035-inch guidewire. 

An 8-Fr sheath was introduced into the left brachial artery (LBA); this was followed by the administration of unfractionated heparin at a rate of 100 U per kilogram, aiming for a target activated coagulation time of 300 s. Subsequently, an MPA1 catheter was guided through the sheath to access the FA. The free end of a traction wire attached to the graft branch was inserted into the MPA1 catheter through the FA and out of the LBA. A super stiff guidewire (specifically, the Lunderquist wire from Cook in Bloomington, IN, USA) was threaded into the ascending aorta using the same femoral access route. The delivery sheath followed the path of the super stiff guidewire until it reached the descending aorta. The delivery system was then rotated to position the branch along the greater curvature of the aortic arch. Subsequently, the stent and the soft inner sheath were carefully advanced into the aortic arch.

An assessment was carried out to verify the correction of the position of the branch and to ensure that the branch traction wire was not tangled. Subsequently, the soft sheath was then retracted using the traction wire to gently guide the branch into the LSA.

For the deployment of the main body, the systolic blood pressure was maintained at approximately 90 mmHg by means of pharmacological intervention. With the position of the stent confirmed under X-ray guidance, deployment of the main body was initiated by pulling the trigger wire. 

Following that, the traction wire was utilized to initiate the deployment of the branch. Shortly after deployment, the patient’s blood pressure was restored to normal levels and angiography was carried out to assess the isolation of the aortic lesion and the patency of the branch. The delivery system and guidewires were then carefully removed.

Closure of the right arterial puncture sites was achieved using ProGlide vascular closure devices. Both the LBA and the contralateral FA were gently compressed for 5 min before applying the bandages. Once the patients had regained consciousness from the anesthesia, they were transferred to the ward for postoperative clinical monitoring.

**Figure 3:**
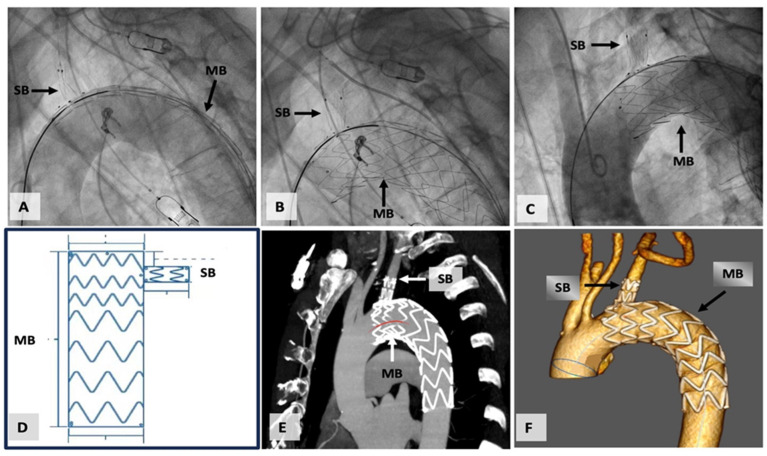
(**A**) Castor single-branched stent graft before its deployment, (**B**) the prosthesis after its deployment, (**C**) angiographic check after prosthesis release, (**D**) schematic diagram of the prosthesis, (**E**) computed tomography check of the correct positioning of the prosthesis, (**F**) three-dimensional volume rendering computed tomography, showing the prosthesis. (MB: main branch; SB: solitary branch).

Subsequent to the surgical procedure, the patient received dual antiplatelet therapy (DAPT) for a duration of 3 months. They were then prescribed a daily dose of 100 mg aspirin for a one-year period. Figure 3 presents the deployment of stent graft and the angiographic and computed tomography checks for the correct positioning of the prosthesis.

### 2.4. Definition of the Clinical Parameters

In the context of this study, early mortality referred to deaths occurring within 30 days after surgery. Diagnosis of a stroke involved identifying cerebral infarction or intracerebral hemorrhage using neuroimaging carried out after a requested neurological evaluation if the patient showed signs or symptoms of stroke. Acute kidney injury was determined based on the Kidney Disease Improving Global Outcomes (KDIGO) guidelines. Endoleaks were defined as the continuous flow of blood outward from the graft or within the aneurysm sac.

### 2.5. Clinical Follow-Up

Following the endovascular repair, postoperative monitoring involved the routine recording of CTA imaging at one, three, and six months post-procedure and subsequently on an annual basis. These scans were carried out to evaluate parameters such as the presence of an endoleak, LSA patency and potential migration, and to assess the remodeling morphologies of dissection or aneurysm.

### 2.6. Statistical Analysis

Statistical analysis was carried out using SPSS version 22.0 (SPSS Inc., Chicago, IL, USA). Patient characteristics, perioperative outcomes, procedural features, and postoperative follow-up were reported using descriptive statistics. Continuous variables were shown as mean (±SD) and median and interquartile range (IQR). The categorical data were expressed as number and percentage.

## 3. Results

### 3.1. Baseline Characteristics

This study involved a group of 10 participants, consisting of 1 female and 9 males, with an average age of 70.6 ± 7.5 years (ranging from 61 to 79 years). The mean body mass index (BMI) of the patients was 27.7 ± 5.2 kg/m². Of these patients, 9 (90%) had hypertension, and 1 (10%) had diabetes mellitus. In the overall cohort, four individuals had a history of tobacco use, three had peripheral vascular disease, two had experienced prior myocardial infarctions, and two had a history of ischemic stroke; none had a history of paraplegia before the surgery. In terms of the types of lesions, three patients had only type B aortic dissection, two had only a PAU, two had only thoracic aortic aneurysms, and one had only an intramural hematoma. In two patients, the coexistence of two types of lesions was found: one patient with aortic aneurysm and dissection, and one patient with PAU and aortic aneurysm. Refer to Table 1 for a detailed breakdown of the demographic and clinical data.

The diameter of the proximal landing zone measured 31.4 ± 2.3 mm, with an oversizing of the proximal stent end by 16% ± 3.2%. At a distance of 25 mm from the ostium, the average diameter of the LSA was 10.7 ± 1.2 mm. In all 10 patients, the proximal graft end was oversized by an average of 15%, and the diameter of the distal LSA was oversized by an average of 12% (see Table 2 for details).

### 3.2. Perioperative Results

Immediately after the surgery, aortograms showed the successful elimination of the lesion and proper perfusion of the LSA in all cases. In addition, a favorable antegrade blood flow in the LCCA was observed at the conclusion of each procedure. The median fluoroscopy time was 28′ 38″ ± 13′ 23″ minutes, and a median contrast volume of 286.4 ± 146.8 mL was utilized. Notably, no patients required admission to the intensive care unit after the operation, and the average hospital stay was 5 days. There were no perioperative mortalities and no serious complications such as stroke, acute myocardial infarction, renal failure, or left arm ischemia occurred (refer to Table 2 for details).

## 4. Follow-Up

The follow-up period averaged one year. Throughout this follow-up period, no adverse events, such as deaths, left arm ischemia, paraplegia, aortic rupture, conversion to open surgery, or the need for secondary endovascular interventions, were reported. There were no instances of stroke or upper extremity ischemia among the patients, and no evidence of stent migration or endoleaks. Aortic remodeling, defined as the postoperative area as a percentage of the preoperative area, was considered positive when the true lumen area was ≥120% with an aortic lumen <120% or when the true lumen area was ≥80% with an aortic lumen <80% [9]. Positive remodeling of the aorta was observed, attributed to thrombosis within the false lumen for aortic dissection cases and thrombosis within the aneurysm lumen for aneurysm cases. Postoperative follow-up CTA imaging confirmed the patency of the single-branched stent grafts (SABs).

## 5. Discussion

The first findings demonstrated that, after an average follow-up of one year, the Castor branching aortic stent graft system was safe, and achieving an appropriate proximal landing zone and maintaining sufficient LSA perfusion was possible; TEVAR is a minimally invasive medical breakthrough therapy that has transformed the treatment of thoracic aortic conditions. Numerous studies have confirmed its effectiveness and safety. In its initial stages, TEVAR received approval from the U.S. Food and Drug Administration for human implantation, specifically for descending thoracic aneurysms located within Ishimaru zones 3–5 [10]. However, ongoing advancements in endovascular stent grafts have extended their application to the proximal descending aorta. A prerequisite for TEVAR is the presence of an adequate proximal landing zone, ranging from 15 to 20 mm on both sides of the aortic lesion [8]. However, the necessity of an adequate proximal landing zone (ranging from 15–25 mm) to obtain a good seal and fixation has sometimes limited its application in the proximal descending aorta. Nevertheless, the utilization of TEVAR has expanded to encompass various lesion types within the context of active employment, driven by ongoing technological advancements. Despite the fact that individuals undergoing endovascular therapy tended to be older, and faced more severe illnesses and additional comorbidities as compared to those opting for open aortic arch repair, the mortality rate among patients undergoing endovascular treatment was significantly lower [10,11].

Thus, more and more TEVAR procedures have been aimed at arch reconstruction for aortic arch aneurysm and aortic dissection [12]. Intentional coverage of the LSA without reconstruction is often performed in patients with an inadequate proximal sealing zone, but can result in serious complications such as stroke and upper extremity ischemia [13,14].

The choices for LSA revascularization include traditional cervical debranching procedures which may involve a carotid-subclavian bypass, a carotid-axillary bypass, or the transposition of the subclavian artery to the carotid artery. As early as 2017, the European Society for Vascular Surgery Guidelines suggested routine revascularization of the LSA in all elective settings [15]. While a carotid-subclavian bypass or transposition (CS-BpTp) is still considered the best option for LSA revascularization in a zone 2 TEVAR setting, it does have some associated risks, such as lymphatic leaks, bleeding, infection, phrenic nerve palsy, and stroke. Deciding between CS-BpTp and endovascular techniques (ETs) requires careful consideration on a case-by-case basis, weighing the peri-operative risks and long-term outcomes. As complications from CS-BpTp can detract from the minimally invasive nature of TEVAR, there is increasing interest in completely endovascular options for LSA perfusion preservation [16]. 

New endovascular techniques have been developed to overcome the LSA revascularization problem. Over the years, two main endovascular techniques have been used in TEVAR for LSA revascularization. In 2007, Criado introduced the chimney technique for aortic endovascular repair; this has since found widespread adoption and is frequently utilized in conjunction with aortic endovascular repair procedures [17]. The use of this technique has been increasingly applied to enhance the proximal landing zone for TEVAR, and it has been documented to yield favorable outcomes [18,19]. Unfortunately, there is notable risk of creating a gutter between the aortic wall and the chimney stent, leading to the frequent occurrence of type Ia endoleaks after TEVAR when employing the chimney technique [20,21].

Another dependable approach in endovascular aneurysm repair is fenestration [22]. In 2004, McWilliams et al. described the in situ fenestration of aortic stent grafts, an off-label technique designed to preserve the LSA during TEVAR procedures [23]. This method appears to be a sensible and effective solution, especially in emergent TEVAR procedures [24]. The primary concern when employing this technique is the potential of the fenestration to propagate and result in a type III endoleak. However, long-term follow-up results for this strategy are being eagerly awaited. Back in 1999, Inoue initially conceptualized a single-branched stent graft to address distal arch diseases involving the LSA. However, this approach yielded a relatively high rate of complications, including endoleaks and cerebral infarctions [25]. While the initial outcomes may not have been very promising due to preoperative complications, the concept of a single-branched stent graft design remains a promising proposition. The unibody design appeared to be able to eliminate the gutter endoleak, the most frequent complication in the chimney technique, and furthermore, seemed to control the risk of a secondary displacement of the graft, owing to the anchoring effect of the branch section, which contributed to stabilizing the entire device. 

The Castor Branched Aortic Stent Graft System, developed by Microport Medical (Shanghai, China), displays several unique design characteristics that set it apart from similar medical devices. To emphasize its distinctive unibody structure, the layout of the system, particularly the proximal positioning of the LSA, is almost orthogonal to the descending aorta. This nearly right-angled setup plays a pivotal role in maintaining the stability of the device within the LSA post-implantation, thus drastically minimizing the risks of device displacement or migration, a common obstacle observed in many similar procedures. Another noteworthy aspect of the Castor system design lies in its diminished frequency of endoleaks post-procedure. A comparative analysis with the more traditional chimney technique demonstrated a significantly lower occurrence of these leaks. This can be largely attributed to the absence of a gutter, generally an unfortunate by-product in instances of single-branched stent graft implantation. With the absence of this gutter in the Castor system design, the propensity for endoleaks is notably reduced, thus ensuring that the overall outcome of the procedure is greatly improved. These specific aspects of the Castor design contribute to its increasing popularity within the medical arena since they effectively address and overcom many of the prevalent challenges observed in these intricate procedures. By offering this unique design solution, the Castor Branched Aortic Stent Graft System effectively sets a new precedent in the field of endovascular interventions.

The authors’ research has revealed that the Castor system is both secure and effective for addressing aortic pathologies during the initial follow-up period. In the present series, an exceedingly low occurrence of significant endoleaks after endovascular aortic arch repair was documented. Despite these positive results, it is essential to recognize that the Castor device is not without limitations. To highlight some specific challenges, there is a notable difference in size when comparing the external diameter of the Castor delivery system to that of straight stent grafts. The increased size calls for a more elaborate intervention process, adding complexity and the potential for complications. Moreover, during the deployment phase of the Castor device, there is an additional risk to consider. The traction wire used in the deployment process has the potential to become entangled with the super stiff guidewire. This could pose a challenge, and requires additional time and skill to navigate, adding another layer of complexity to the procedure.

Finally, it is worth noting that applying the Castor system is not universally convenient or immediate due to its custom-made nature. The time it takes for the planning and delivery of this individualized device usually spans a couple of weeks, restricting its usage exclusively to cases that allow for an elective setting. The inherent lead time of the Castor system means that it cannot be used for on-demand or immediate procedures. As such, this limitation reduces the applicability and flexibility of the system in some diverse clinical scenarios.

## 6. Limitations

This study has several limitations, including a relatively small sample size, a brief follow-up duration, and the absence of a control group. Subsequent research should aim to compare and contrast the clinical outcomes of this endovascular branched stent graft with established methods, such as open surgery, hybrid procedures, and chimney techniques.

## 7. Conclusions

Based on the authors’ limited experience, the utilization of the Castor branched aortic stent graft system was proven to be both safe and practicable in securing an adequate proximal landing zone while preserving the required LSA perfusion over an average follow-up period of one year. This product offers an appealing alternative for addressing distal aortic arch pathologies by means of endovascular repair. However, to comprehensively assess the long-term effectiveness of this technique, it is essential to extend the follow-up duration.

## Figures and Tables

**Table 1:** Patient characteristics before stent graft implantation for the endovascular repair of the thoracic aorta.

Baseline Clinical Characteristics	
Age (median IQR)	76 61–79
BMI (median IQR)	29.7 20.6–34.5
Male (n; %)	9; 90%
Smoker (n; %)	4; 40%
Arterial hypertension (n; %)	9; 90%
Diabetes mellitus (n; %)	1; 10%
Dislipidemia (n; %)	8; 80%
Hb (g/dL) pre (median IQR)	13.4 11.9–15.6
Previous Myocardial Infarction (n; %)	2; 20%
Previous stroke (n; %)	2; 20%
Carotid artery disease (n; %)	2; 20%
Lower limb arterial disease (n; %)	1; 10%
Ejection fraction (%) (median IQR)	58 41–65
**Indications**	
Type B Aortic dissection (n; %)	4; 40%
Intramural haematoma (n; %)	1; 10%
Penetrating aortic ulceration (n; %)	3; 30%
Aortic aneurysm (n; %)	4; 40%
LSA pathology (re-entry, aneurysm) (n; %)	0; 0%

BMI: body mass index; Hb: hemoglobin; LSA: left subclavian artery.

**Table 2:** Anatomical, procedural features, and short- and mid-term complications. (LSA: left subclavian artery).

Preoperative CTA measurements		
Proximal landing mm (Median IQR)		31.4 27–35
Distal landing mm (Median IQR)		32.1 26.0–40
Proximal oversizing mm (Median IQR)		5 3–7
Distal LSA mm (Median IQR)		10.7 9.5–12.9
LSA oversizing mm (Median IQR)		1.3 0–2.5
Arch type (n; %)	I	1; 10%
	II	3; 30%
	III	6; 60%
	**Procedural data**	
Fluoroscopy time (Median IQR)		26′ 1″ 17′ 9″–34′ 1″
Contrast medium (mL) (Median IQR)		286.4 120–580
General Anesthesia (n; %)		7; 70%
Surgical access (n; %)		1; 10%
Hospitalization (Median IQR)		4.3 2–10
Percutaneous access (n; %)		9; 90%
Technical succes (n; %)		10; 100%
**Short and Mid-term complications**		
Bird Beak (n; %)		0; 0%
Endoleak Tvpe 1 (n; %)		0; 0%
Endoleak Type 2 (n; %)		0; 0%
Endoleak Type 3 (n; %)		0; 0%
Intraoperative complications (n; %)		0; 0%
Intraoperative cardiac arrest (n; %)		0; 0%
Intraoperative death (n; %)		0; 0%
**Preoperative CTA measurements**		
Proximal landing mm (Median IQR)		31.4 27–35
Distal landing mm (Median IQR)		32.1 26.0–40
Proximal oversizing mm (Median IQR)		5 3–7
Distal LSA mm (Median IQR)		10.7 9.5–12.9
LSA oversizing mm (Median IQR)		1.3 0–2.5

## Data Availability

The data regarding this article will be shared on reasonable request to the corresponding authors. The data are not publicly available due to privacy.
